# Incidence, risk factors and clinical course of pyogenic spondylodiscitis patients with pulmonary embolism

**DOI:** 10.1007/s00068-021-01776-z

**Published:** 2021-09-02

**Authors:** Daniel Dubinski, Sae-Yeon Won, Fee Keil, Bedjan Behmanesh, Max Dosch, Peter Baumgarten, Joshua D. Bernstock, Volker Seifert, Thomas M. Freiman, Florian Gessler

**Affiliations:** 1 Department of Neurosurgery, University Hospital, Goethe University, Schleusenweg 2-16, 60598 Frankfurt, Germany; 2 Institute of Neuroradiology, University Hospital, Goethe University, Frankfurt, Germany; 3 Department of Neurosurgery, Brigham and Women’s Hospital, Harvard Medical School, Boston, MA USA

**Keywords:** Pulmonary embolism, Pyogenic spondylodiscitis, Risk factor, Coronary heart disease

## Abstract

**Purpose:**

In patients with pyogenic spondylodiscitis, surgery is considered the treatment of choice to conduct proper debridement, stabilise the spine and avoid extended bed rest, which in turn is a risk factor for complications such as deep vein thrombosis and pulmonary embolism.

**Methods:**

We conducted a retrospective clinical study with analysis of a group of 99 patients who had undergone treatment for pyogenic discitis at our institution between June 2012 and August 2017. Included parameters were age, sex, disease pattern, the presence of deep vein thrombosis, resuscitation, in-hospital mortality, present anticoagulation, preexisting comorbidities, tobacco abuse, body mass index, microbiological germ detection and laboratory results.

**Results:**

Among the analysed cohort, 12% of the treated patients for pyogenic spondylodiscitis suffered from a radiologically confirmed pulmonary embolism. Coronary heart disease (*p* < 0.01), female sex (*p* < 0.01), anticoagulation at admission (*p* < 0.01) and non-O blood type (*p* < 0.001) were associated with development of pulmonary embolism. Pulmonary embolism was significantly associated with resuscitation (*p* < 0.005) and deep vein thrombosis (*p* < 0.001). Neurosurgery was not associated with increased risk for pulmonary embolism compared to conservative-treated patients (*p* > 0.05).

**Conclusion:**

Surgery for pyogenic spondylodiscitis was not associated with an elevated risk of pulmonary embolism in our analysis. However, we describe several risk factors for pulmonary embolism in this vulnerable cohort. Prospective studies are necessary to improve prevention and postoperative management in patients with pyogenic spondylodiscitis.

## Introduction

Pyogenic spondylodiscitis is a rare disease with high morbidity and consecutive long-term sequelae [1]. The bacterial infection is usually of hematogenic origin and transmitted from the oral cavity, the respiratory tract or the skin [2, 3]. The primary site of infection is the avascular disc, the infection may then spread continually into the vertebral body and up into the subligamentous paravertebral area, epidural space and contiguous vertebral bodies. In consequence, back pain and limitation of spine movement are the predominant signs in patients with spondylodiscitis [4, 5]. Treatment concepts of pyogenic spondylodiscitis include either a conservative or a surgical management and should be evaluated individually. The currently available therapeutic guidelines are not standardised and based on individual preferences resulting in a high variability of outcome with conflicting results [6–8].

The conservative treatment consists of germ-compatible long-term antibiotic treatment and immobilisation of the spine [5, 9]. In patients with progressive neurologic deficits, large abscesses, progressive deformities or involvement of at least two adjacent vertebral bodies, surgical management should be favoured [10]. Surgical strategies are versatile and include extensive debridement of the disc and vertebral bodies, stabilisation and titanium mesh cages [10, 11]. Hence, patients treated for pyogenic spondylodiscitis harbour a numerous amount of well-established risk factors for pulmonary embolism such as immobilisation and prolonged bed rest, infection and bacteremia, operation and neurological deficits such as paralysis or paraplegia [12, 13]. The currently lacking literature on patients with pyogenic spondylodiscitis and pulmonary embolism in the scientific literature prompted this study. Per our hypothesis, patients with pulmonary embolism may accommodate risk factors that are currently unknown to the literature. The identification of such risk factors may contribute to future concepts for thromboprophylaxis in this demanding cohort.

## Methods

### Study design

The present analysis is a retrospective, single centre observational study of patients with pyogenic spondylodiscitis. The hypothesis of the study was that several, to date largely unknown, risk factors contribute to the development of pulmonary embolism in patients with pyogenic spondylodiscitis. The identification of these could lead to a better risk stratification and adjusted anticoagulation regimes in this vulnerable cohort and potentially improve outcome.

### Patients and data collection

For this retrospective analysis, an ethical approval was obtained by the local ethics committee (identification number: 20-683). As a non-interventional single-centre study no patient consent was necessary. Patients over 18-years old who were surgically or conservatively treated for pyogenic spondylodiscitis from 2012 to 2017 were identified retrospectively. Diagnosis of pyogenic spondylodiscitis was made upon clinical evaluation and spinal MRI with contrast agent. Indication for surgery was based upon individual case by case discussion including patients will, neurologic deficit, large abscesses, or involvement of at least two adjacent vertebral bodies. Surgery and postoperative management was performed as described before [14, 15]. Indication for thoracic CT scan was acute onset of one, or the combination of the following symptoms: collapse upon mobilisation, shock, hypotonia, tachycardia, dyspnoea, chest pain or dip in oxygen saturation [16]. Patients with a pre-existing haematological disorder (e.g. factor V Leiden, prothrombin mutation, protein C/S deficiency, leukaemia, lymphoma, systemic amyloidosis) were excluded. Further exclusion criteria were treatment of patient for less than 48 h. For venous thromboembolism (VTE) prophylaxis, all patients received s.c. low molecular-weight heparin (LMWH) within 48 h of admission. In patients with elective surgical procedures, phenprocoumon therapy was paused at least 2 weeks prior surgery and switched to therapeutic LMWH which was paused on the day of surgery. The primary outcome was in hospital mortality. All patients received 40 mg of LMWH (Clexane^®^) subcutaneously starting on the first postoperative day. On the day of operation, 20 mg of LMWH (Clexane^®^) were administered s.c. 10 h post-surgery. This anticoagulation concept was recommended by our department of hemostaseology and applied in our department for over 20 years. Mobilisation was anticipated as soon as possible and realised with the assistance of a physiotherapist. All patients were urged to wear compression stockings. Patients were seen in a follow-up examination 3-months post-surgery in our out-patient department.

### Computed tomography imaging

Thoracic CT scans were performed in the department of neuroradiology at a multidetector Philips CT Scanner. Ultravist^®^ 300 was administered intravenously (80 ml/kg, 4.0 ml/s) and imaging started after the contrasting of the pulmonary artery.

### Spinal MRI

All patients received spinal MRI in the department of neuroradiology, Goethe University Hospital Frankfurt at a 3 Tesla Siemens Verio scanner. Gd‐DO3A‐butrol (Gadovist^®^, Bayer Vital GmbH) was administered intravenously (0.1 ml/kg; max. 10 ml). T1w sagittal post contrast imaging started directly, axial T1w post contrast imaging was preformed between 3 and 7 min after administration of the contrast agent.

### Statistics

Data analysis was performed with IBM SPSS Statistics Version 23.0 (SPSS Inc., IBM Corp., Armonk, NY, USA). For patient characteristics, descriptive statistics were used. Fisher’s exact test was used for the comparison of categorical variables between the cohorts. For continuous parameters, the Wilcoxon–Mann–Whitney test was used. To assess the impact of the variables, odds ratio (OR) with 95% confidence intervals (CI) was calculated. Results with *p* ≤ 0.05 were considered statistically relevant.

## Results

### Cohort characteristics

**Fig. 1:**
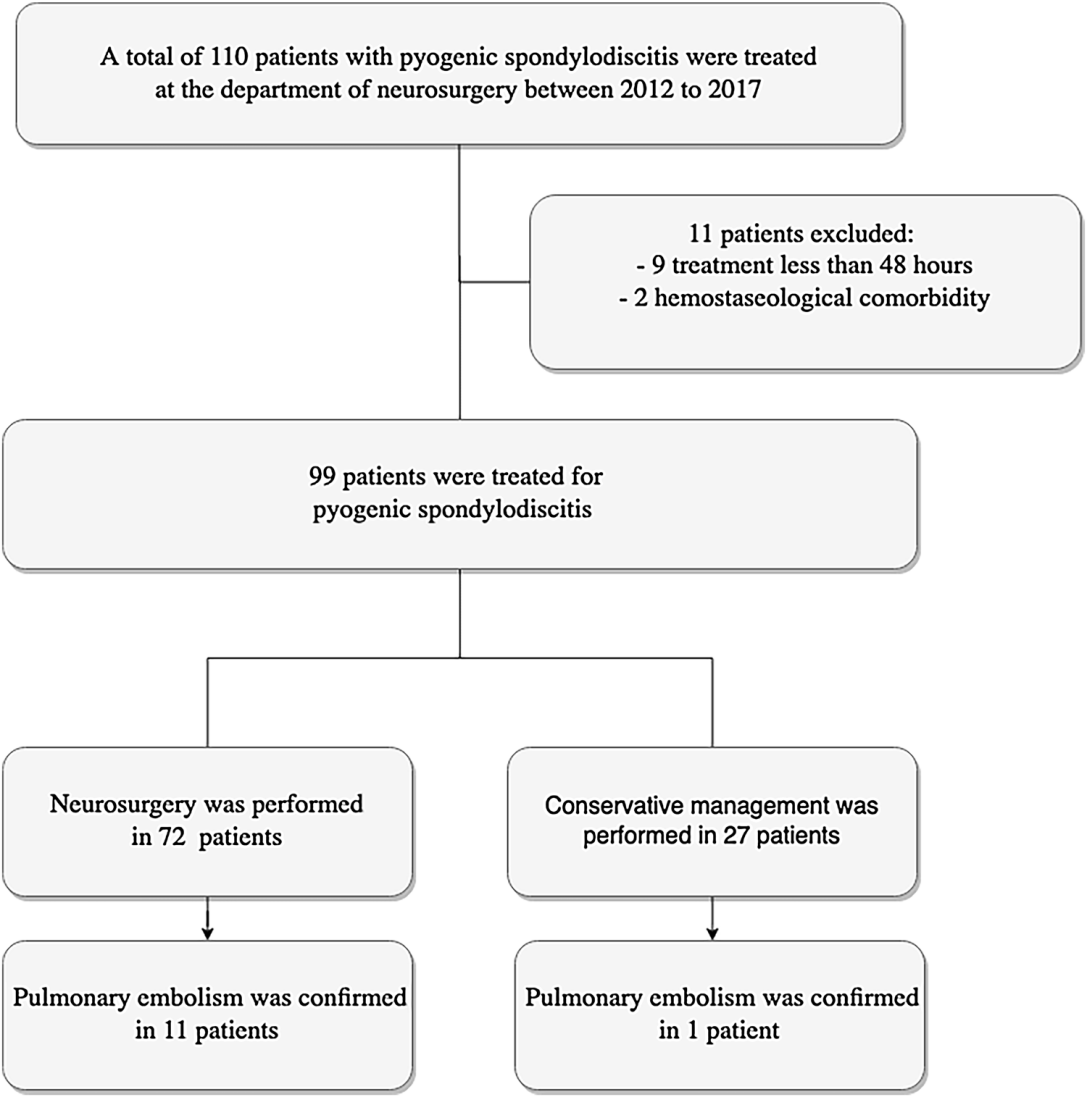
Flow diagram displaying an overview of the patients included for analysis

A total of 110 patients with treatment of pyogenic spondylodiscitis were included. The observational time frame was 2012–2017. In total, two patients were excluded from the study due to a pre-existing hemostaseological burden and nine due to treatment less than 48 h. Hence, a total of 99 patients with pyogenic spondylodiscitis were included in the analysis. A flow diagram is displayed in Fig. 1.

Overall, 59 patients (60%) were male and median age was 75.5 (SD: 10.1) in the analysed cohort. The site of infection was distributed as following: cervical spine 12 (12%) patients, thoracic spine 33 (33%) patients and 54 (55%) patients with spondylodiscitis of the lumbar spine. Neurosurgical intervention was performed in 72 cases (72%). In detail, decompression was done in 18 patients (25%), dorsal or ventral approach in 28 (39%) and combined dorsoventral approach in 23 (32%).

A pathogen detection could be obtained in 45 patients (45%). In total, 51 patients had non-O blood type (52%). In total 12 patients (12%) suffered from postoperative PE. Resuscitation (defibrillation, chest compression, intubation and pharmacological intervention) was necessary in four patients (4%). Deep vein thrombosis was confirmed in two patients (2%) and four patients died during the hospital stay (4%). Four patients had a known history of tobacco abuse (4%) and obesity was observed in 21 patients (21%). Anticoagulation at admission (phenprocoumon, LMWH, platelet aggregation inhibitors or direct oral anticoagulants) was present in 24 patients (24%), coronary heart disease was present in 16 patients (16%) and hypertension in 32 (32%) of the analysed cohort. Diabetes mellitus was observed in 26 (26%) patients and four patients (5%) were on antidepressants at admission. See Table 1.

**Table 1:** Patient characteristics (*n* = 99)

	*n* (%)
Sex	
Male	59 (60)
Female	40 (40)
Age (SD)	75.5 (10.1)
Location	
Cervical spine	12 (12)
Thoracic spine	33 (33)
Lumbar spine	54 (55)
Neurosurgery	
Yes	72 (72)
No	27 (28)
Neurosurgical approach (*n* = 72)	
Decompression	18 (25)
Dorsal or ventral approach	28 (39)
Dorsoventral approach	23 (32)
Laboratory results	
Proof of germination	45 (45)
Non-O blood type	51 (52)
Pulmonary embolism	
Yes	12 (12)
No	87 (87)
Morbidity and mortality	
Resuscitation yes	4 (4)
Resuscitation no	95 (96)
Deep vein thrombosis (DVT) yes	2 (2)
Deep vein thrombosis (DVT) no	97 (98)
Outcome	
Death	4 (4)
Survival	95 (96)
Risk factors	
Tobacco abuse yes	4 (4)
Tobacco abuse no	95 (96)
Obesity yes	21 (21)
Obesity no	78 (78)
Pre-existing comorbidities	
Coronary heart disease yes	16 (16)
Coronary heart disease no	83 (84)
Hypertension yes	32 (32)
Hypertension no	67 (68)
Diabetes mellitus yes	26 (26)
Diabetes mellitus no	73 (74)
Medication at admission	
Anticoagulation yes	24 (24)
Anticoagulation no	75 (76)
Antidepressants yes	5 (5)
Antidepressants no	94 (95)

*SD* standard deviation

#### Risk factors for pulmonary embolism in pyogenic spondylodiscitis

Female sex was significantly associated with PE (*p* < 0.01). The median age in the PE positive group was 82 (SD: 10) and 76 (SD: 14.6) in patients without PE. Patient age was not associated with PE development (*p* = 0.17). We further investigated the possible impact of the infection site on PE development. In total, two patients (17%) with PE had pyogenic spondylodiscitis of the cervical spine and 9 (11%) of the patients without PE cohort (*p* = 0.35). The thoracic spine was affected in three patients (28%) in the PE cohort and 24 patients (27%) in in patients without PE, and was therefore, not significantly correlated (*p* = 0.46). Lumbar spine was primary infected in seven patients in the PE cohort (58%) and 54 patients (62%) of patients without PE and therefore not significantly associated (*p* = 0.59). Neurosurgical intervention was performed in 72 cases in which 11 patients developed PE vs. a total of 26 patients with conservative treatment in which one patient developed PE. Neurosurgical intervention was therefore not statistically significant in PE development (*p* = 0.17).

In detail, in patients who underwent spinal decompression seven patients (64%) developed PE vs 23 (38%) without PE detection. In patients with dorsal or ventral approach 2 (18%) patients developed PE vs 17 (28%) without PE development. Furthermore, in patients with combined dorsoventral approach, two patients (18%) developed PE vs. 21 (34%) without PE. The operative approach, was therefore, not significantly associated with PE (*p* = 0.18); (*p* = 0.71) and (*p* = 0.32), respectively.

The proof of germination (Blood cultures and/or operative smear) was achieved in 7 (63%) patients in the PE cohort and 28 (46%) in the control group (*p* = 0.18). All patients (*n* = 11) had non-O blood type in the PE cohort and 26 (42%) in the control group. Patient blood type was significantly associated with PE risk (*p* = 0.001) (Table 2).

**Table 2:** Univariate analysis of patients with pyogenic spondylodiscitis with radiographically confirmed or unconfirmed pulmonary embolism as the dependent variable

Patient characteristics	Pulmonary embolism		Univariate analysis		
	Yes (*n* = 12)	No (*n* = 87)	OR	95% CI	*p* value
Sex					
Female, (%)	9 (75)	32 (37)	5.89	1.40–24.71	**0.01**
Age, (SD)	82 (10)	76 (14.6)	n/a	2.84–14.82	**0.17**
Location					
Cervical spine, (%)	2 (17)	9 (11)	2.03	0.35–11.70	0.35
Thoracic spine, (%)	3 (25)	24 (27)	0.71	0.17–2.97	0.46
Lumbar spine, (%)	7 (58)	54 (62)	0.95	0.26–3.56	0.59
No neurosurgical intervention, (%)	1	26	0.21	0.02–1.73	0.17
Neurosurgery, (%)	11	61			
Neurosurgical approach (*n* = 72)					
Decompression, (%)	7 (64)	23 (38)	2.89	0.76–10.96	0.18
Dorsal or ventral approach, (%)	2 (18)	17 (28)	0.57	0.11–2.93	0.71
Dorsoventral approach, (%)	2 (18)	21 (34)	0.42	0.08–2.14	0.32
Laboratory results					
Proof of germination, (%)	8 (66)	41 (47)	2.24	0.62–8.00	0.23
Non-O blood type, (%)	12 (100)	39 (44)	n/a	0.31–0.57	**0.001**
Morbidity and mortality					
Resuscitation, (%)	3 (25)	1 (1)	28.6	2.69–305.15	**0.005**
Deep vein thrombosis, (%)	3 (25)	0 (0)	n/a	n/a	**0.001**
Outcome					
Death, (%)	1 (8)	0 (0)	n/a	n/a	0.12
Risk factors					
Tobacco abuse, (%)	1 (9)	5 (6)	1.4	0.15–13.96	1
Obesity, (%)	2 (17)	15 (17)	0.96	0.19–4.83	1
Pre-existing conditions					
Coronary heart disease, (%)	ä	9 (10)	6.19	1.62–23.62	**0.01**
Hypertension, (%)	6 (50)	21 (24)	3.14	0.91–10.78	0.08
Diabetes mellitus, (%)	2 (17)	19 (21)	0.71	0.14–3.54	1
Medication at admission					
Anticoagulation, (%)	6 (50)	14 (16)	5.21	1.46–18.52	**0.01**
Antidepressants, (%)	1 (8)	5 (6)	1.49	0.15–13.96	1

*OR* odds ratio, *SD* standard deviation

*p* = 0.001

Furthermore, anticoagulation at admission was present in six patients (50%) in the PE cohort vs. 14 (16%) in the PE negative cohort. The presence of anticoagulation at admission was therefore statistically significant associated with PE development (*p* = 0.01).

### Acquired risk factors

Tobacco abuse was observed in one patient in the PE group (9%) and five patients in the PE negative cohort (6%), and was therefore, not associated with PE (*p* = 1). Obesity was present in two patients in the PE cohort (17%) and 15 (17%) in patients without PE detection, and was therefore, not significantly associated (*p* = 1). See Table 2.

### Pre-existing comorbidities

Coronary heart disease was present in five patients (42%) with PE and nine patients (10%) without PE development. The presence of coronary heart disease was significantly associated with PE onset (*p* = 0.01). Further, hypertension was present in six patients (55%) in the PE group and 21 patients (24%) in the PE negative cohort (*p* = 0.08). Diabetes mellitus was present in two patients (17%) in the PE cohort and in 19 patients (21%) without PE development. The presence of diabetes mellitus, was therefore, not associated with PE onset (*p* = 1). See Table 2.

### Morbidity and mortality

Resuscitation was performed on three patients (25%) in the PE cohort and in 1 (1%) patient without PE development, and was therefore, significantly correlated (*p* = 0.005). Deep vein thrombosis was confirmed in three patients (25%) in the PE group and no patient without PE development and was significantly associated with the diagnosis of PE (*p* = 0.001). One patient died in the PE cohort (8%); whereas, none of the patients in the PE negative cohort. The in-hospital mortality, was therefore, not significantly associated with PE *p* = 0.12. See Table 2.

## Discussion

To the best of our knowledge the present study is the first to describe the remarkably high incidence of PE in patients with pyogenic spondylodiscitis compared to published numbers of other surgical disciplines [17–19]. Furthermore, we identified female sex, higher age, coronary heart disease, anticoagulation at admission and non-O blood type as significant risk factors for PE. PE was associated with DVT and resuscitation. Surprisingly, neither surgical intervention itself nor the chosen approach were associated with elevated PE risk compared to conservatively treated patients.

In our cohort, 12% of the included patients with pyogenic spondylodiscitis suffered from PE. This incidence appears to be significantly higher than described in the scientific literature. As for example studies on PE after orthopaedic surgeries, including a large series of spinal surgeries, vary from 2 to 6% [16, 20, 21]. However, the published variety of PE manifestation among spinal surgery is extremely high as other studies report an incidence of up to 31% (i.e. patients with major spinal reconstruction) [17, 18]. Our finding therefore amends to the published knowledge and raises the question of preventive scopes of action and anticoagulation management in patients with surgical interventions for pyogenic spondylodiscitis and those with conservative management.

The finding of significantly overrepresented female patients in the PE cohort is intriguing as data on whether patient sex is a risk factor for pulmonary embolism is conflicting. An analysis of national mortality data found that death rates from PE were up to 30% higher among men than among women [21]. Although female sex was not found to be a significant risk factor in large unselected observational studies, female patients with PE had higher in-hospital mortality, a higher need for transfusion and occurrence of shock [22].

Further, all patients in our PE cohort had non-O blood type. The difference in hemostaseological properties of ABO antigens led to the identification of non-O blood type as a significant risk factor for postoperative thrombosis and embolism [23, 24]. Our finding is in line with this observation and demonstrates a significant association of patient blood type and for postoperative PE in patients with pyogenic spondylodiscitis.

Several publications claim that patients with high BMI are at elevated risk for PE [19]. In specific, excessive visceral adipose tissue causes hypoxia and increases free fatty acids (FFA) to the liver where coagulation factors are synthesised [25]. However, our study found no association between patients BMI and PE. A possible explanatory approach is the fact that in our analysis the investigated BMI was obtained at admission. Since pyogenic spondylodiscitis is a disease of mid- to long-term duration, patient BMI was subject to fluctuations after admission and thus not detected in our analysis.

In our cohort, the presence of anticoagulation at admission was associated with PE. This finding is challenging. All patients on anticoagulation were converted to LMWH prior to neurosurgery as described in the methods section. In most cases, the rational for anticoagulation was a positive history for DVT or PE. The underlying coagulation disorder that led to DVT or PE in the first place could therefore be accountable for PE in our cohort. Furthermore, a reactive hypercoagulability state or an increased hemostaseological response could also be made accountable for this association. The American College of Chest Physicians (ACCP) and the Eastern Association for the Surgery of Trauma (EAST) have recommended the use of low‐dose unfractionated heparin (UFH) or low molecular weight heparin (LMWH) with or without mechanical prophylaxis for the prevention of VTE complications but they are unclear on timing its initiation [26, 27]. However, we here described our individual approach of administering 20 mg LMWH 10 h postoperatively and report a satisfactory result in postoperative management since our surgically treated patients did not show an increased PE development compared to the conservatively treated.

We found coronary heart disease to be significantly associated with PE in our cohort. Although, we are not aware of other studies that explored the association between pyogenic spondylodiscitis and PE, the increased risk of PE after the diagnosis of coronary heart diseases is known in literature and explained by the fact that coronary heart diseases induce venous stasis and elevated systemic venous pressure [28, 29]. In our cohort, this phenomenon could be aggravated by the present bacteremia, with often infective endocarditis and consecutive further deterioration [30].

In our cohort, patients with PE had a significant association with DVT and resuscitation. The complex situation of a hazardous cardiovascular event with often inevitable long-term anticoagulation after a recent neurosurgical procedure is a known risk factor for poor outcome and coherent with the scientific literature [20, 23].

Furthermore, we found surgical intervention for pyogenic spondylodiscitis to be a non-significant risk factor for PE when compared to a conservative treated cohort. This finding is surprising since major surgery is a well-established risk factor for PE development in the scientific literature [31–33]. The complexity and long duration of both conditions could be accountable for this finding. Hypothetically, surgery could be accountable for a periodic hypercoagulability state with increased thrombotic potential but the benefit of early mobilisation could counterbalance this finding when compared to prolonged bed rest in conservative managed patients.

The obvious limitation is that this investigation was a single centre study and of retrospective design. As this study is of observational character, confounding, selection bias, reverse causation and uncontrolled statistical error risk cannot be excluded. Further, the small sample size of our PE cohort is a possible confounder which should be addressed in future prospective multicentre studies. Despite the relative high incidence of PE in our surgical cohort, when compared to conservative treated patients the sequela is non-hazardous. Only one patient died in the PE cohort. In contrast, data from patients with spinal cord injury display a DVT incidence of almost 100% [34, 35]. The benefits of surgical treatment in patients with pyogenic spondylodiscitis (bacterial detection, reduced bed rest and early ambulation) outweighs the incidence of PE. Because of the small sample a size specific interaction between the variables measured by a multivariate analysis was not possible. Ultimately, a randomised clinical trial is necessary to verify our findings.

## Conclusion

Evidence provided by our study may improve our understanding of the risk factors for PE associated with the treatment of patients with pyogenic spondylodiscitis and may potentially lead to improved prevention and postoperative management.
